# Child Nutritional Status: A Representative Survey in a Metropolitan School

**DOI:** 10.1155/2013/395671

**Published:** 2013-01-28

**Authors:** Paolo Rosati, Stefania Triunfo, Giovanni Scambia

**Affiliations:** Department of Obstetrics and Gynecology, Catholic University of Sacred Heart, Largo A. Gemelli 8, 00168 Rome, Italy

## Abstract

*Objective*. To assess the prevalence of obesity, overweight, and thinness among children in an Italian school. *Methods*. Five hundred ninety-five children (289 males and 306 females) were enrolled, aged between 6 and 19 years old, in Italian school in Rome. Body mass index (BMI) was calculated according to International Obesity Task Force (IOFT) cut-off points. By age criterion all participants have been classified in age classes. *Results*. A normal BMI was recorded in 73.6% of all cases. Obesity, overweight, and thinness prevalence was 5.9%, 9.6%, and 10.9%, respectively, without statistical differences in both genders, except the prevalence of overweight that resulted statistically significant (13.1% males versus 6.2% females, *P* < 0.05). Differences in the age groups have been found. About 23.4% of children between 7 to 11 years were defined obese and about 42.3% between 6 to 8 years thin grade 2, respectively. *Conclusion*. The study reports the low prevalence of overweight and obesity, in contrast to the unexpected thinness prevalence. The identification of specific age groups with abnormal nutritional status could be the first step to address future epidemiological investigations in order to plan strategic approach in selected age periods.

## 1. Introduction

The prevalence of abnormal nutritional status has increased in children and adolescents since 1980 in both developed and developing countries [1], alerting the public health system for the possible risks in terms of cardiovascular, metabolic, and psychiatric diseases [2–5] and economic impact for additional costs to prevent or manage the illness [6]. 

Obesity, overweight, and thinness are variously distributed in the world, in preschool and school children and in both genders over the years. World Health Organization (WHO) estimated that the prevalence of children <5 years of age with a BMI > 2 SD (equivalent to the 98th percentile) increased from 4.2% in 1990 to 6.7% in 2010 and is expected to reach 9.1% in 2020, higher in developed (11.7%) rather than developing countries (6.1%) [7]. In USA the obesity prevalence has increased from 7% in 1980 to nearly 20% in 2008 in children aged 6–11 years and from 5% to 18% over the same period in adolescents aged 12–19 years, respectively [8]. In European countries and regions wide variations in overweight and obesity prevalence estimates among primary-school children have been reported, suggesting the presence of a north-south gradient with the highest level of overweight found in southern European countries [9–11].

Surprisingly, after the global alert for overweight and obesity in infants, children, and adolescents, an opposite extreme on the same spectrum of malnutrition status has drawn attention in the developed countries, especially. Considering the malnutrition as “undernutrition,” the first difficult relieved was its classification, appeared unsatisfactory because of the lack of suitable cut-offs for international use [12]. The reference levels proposed by Cole et al. should helped to provide internationally comparable prevalence rates of thinness in children and adolescents [13]. Actually, however, paucity of data about the thinness in childhood has been recorded [14, 15], frequently associated to anorexia nervosa identified as the third most common chronic condition of adolescence [16].

The aim of the present study, defined as pilot study, was to assess the obesity, overweight, and thinness prevalence in specific age groups in an Italian scholastic population and to plan subsequent surveys in order to precise the target age groups, susceptible of health educational programs. 

## 2. Methods

A cross-sectional 2009-2010 school-year survey among children and adolescents, aged between 6 and 19 years, in Rome was performed. The school was chosen both for the interest to participate in the screening program and for the definition of “Italian scholastic population type” by authors. From an initial sample of 623 students, a final study population of 595 subjects (95.5%) has been enrolled: 15 students (2.4%) were absent for illness or personal reasons and 13 (2.1%) refused to participate, respectively. All parents provided an informed written consent for children in the absence of majority. 

Height and weight were evaluated with standardized protocols and calibrated equipment during a physical examination in the appropriate nursing school. Decimal age was calculated from the data of birth considering the date of measurement. Weight was measured with an electronic SECA bathroom scale previously calibrated to ±0.1 kg. Children were weighed without shoes, jumpers, or sweatshirts and weight recorded to the last 0.1 kg. Height was measured to the nearest 0.1 cm using a Holtain's stadiometer. 

BMI was calculated as weight in kilograms divided by height in meters squared (kg/m^2^). BMI classes were calculated according to the IOFT, identifying these criteria as the most stringent of all the current definitions of childhood obesity [13, 17]. According to WHO that recognizes “thinness” as low BMI for age in adults and adolescents, we used the same definition and not “underweight.” BMI values below 18.5 were distributed into two grades: grade 1 thinness (BMI between the 17 and <18.5 cut-offs, from the 3rd to the 16th centile) and grade 2 thinness (BMI between the 16 and the 17 cut-offs, from the 0.6th to the 3rd centile) [18]. In consideration of absence of cases, cut-offs values < 16, used for grade 3 thinness, were not considered. The data obtained were divided into groups according to age and sex.

Statistical analysis was performed using a chi-square test. *P* value  ≦0.05  was considered significant.

## 3. Results

The sample of 595 children included 306 girls (51.4%) and 289 boys (48.6%), as reported in Table 1. Four hundred thirty-eight children (73.6%) had a normal BMI between the 88th and 16th percentile, and 92 (15.5%) were overweight (57 cases, 9.6%) or obese (35 cases, 5.9%), respectively. The prevalence of thinness was 10.9% (65 cases): grade 1 was detected in 29 cases (4.9%) and grade 2 in 36 (6%), respectively. Prevalence of obesity and thinness grades 1 and 2 were not different significantly in males and females (6.2% versus 5.6%, 4.8% versus 4.9%, and 5.6% versus 6.5%, resp.). The prevalence of overweight was statistically significant between genders (13.1% males versus 6.2% females, *P* < 0.05). 

In relation to the childhood age, the overweight was higher in females between 9 and 13 years (18%) than in males, with an almost equal distribution in all age groups. The highest value in obesity prevalence was found in the 7–11 years age group. In these age groups, 23.4% of children were considered obese, without differences between both genders. Severe thinness was recorded in age groups between 6 and 8 years (42.3%), without difference between genders.

## 4. Discussion

Our study demonstrates that the abnormal nutritional status in children and adolescents represents a considerably larger public health problem, not only for the increase of overweight and obesity prevalence, but also the thinness condition in selected age groups.

Previous studies have shown the prevalence in all countries, the trends over the years, and the impact of both immediate and long-term effects on health and well-being, increasing the risk of cardiovascular and metabolic diseases in adulthood [8, 19–27]. Much has been written about its genetic, nutritional and obstetric risk factors [23, 26, 28, 29]. Profound insights into the biological regulation of appetite, food intake, and weight gain have been gained by identifying and characterizing the rare genetic mutations in individuals and families with extreme obesity, especially [30]. Attention has been given to maternal weight gain in pregnancy, concluding that increased adiposity at birth may predispose to increased risk of obesity and highlight the importance of the impact that women avoid gaining excessive weight in pregnancy [29]. Preventive and treatment strategies have been proposed, recognizing that the childhood malnutrition is not an issue for the education sector alone, but it needs to be tackled at a multisectorial level, identifying the particularly important role of local governments, nongovernment organizations, and the media [31].

In relation to the assessment of nutritional status in Italian children recent studies founded that the excess weight concerns one child out of four, in association to significant differences in the prevalence of overweight and obesity in relation to geographical distribution (Northern, Central and Southern Italy) [32]. Recently, an important survey performed by Italian Ministry of Health has achieved that 23.6% of all Italian children are overweight, while 12.6% obese at 8 years old [33]. However, in the most studies an accurate distribution of all scholastic population by age is not given. Although our study population is lower than previously described (595 versus 2610 cases), the strength of this study is the distribution of all students from 6 to 19 years old by age in order to identify all specific age groups requiring an appropriate health program.

In consideration of the age distribution of our study population, identifyed as another strength of our investigation, we can detected the prevalence of thinness, commonly neglected for the greater impact of obesity and overweight on well-being. Surprisingly, in developing countries the undernutrition is growing, in females particularly. Obviously, we evaluated our results considering the different growth rate in both genders. Adjustments for growth time are important, as recommended by WHO [18], because female overweight is strongly associated with early growth, while the same time is related to low BMI in male gender [34]. Trend of early maturation is recognized recently [17], and evidence is reported in terms of prevalence of thinness in male group and overweight in female group, respectively [34].

In conclusion, nutritional status should be considered in specific age groups and, if confirmed in future and larger studies, the high degrees of malnutrition condition (obesity and thinness) are relieved in target age groups. If our initial findings will be confirmed on a larger population, therapeutic approach to specific age groups can be planned in order to achieve long-lasting benefits.

## Figures and Tables

**Table 1:** Correlation between age groups and BMI.

				Thinness grade 2				Thinness grade 1				Overweight				Obesity			
Age (years)	*n*. (total)	Girls *n*.	Boys *n*.	Girls		Boys		Girls		Boys		Girls		Boys		Girls		Boys	
				*n*.	%	*n*.	%	*n*.	%	*n*.	%	*n*.	%	*n*.	%	*n*.	%	*n*.	%
6 < 7	41	23	18	8	34,8	10	55,6	1	4,3	1	5,6	1	4,3	0	0,0	3	13,0	0	0,0
7 < 8	37	22	15	10	45,5	5	33,3	0	0,0	0	0,0	0	0,0	1	6,7	5	22,7	2	13,3
8 < 9	26	13	13	0	0,0	1	7,7	0	0,0	0	0,0	0	0,0	0	0,0	4	30,8	3	23,1
9 < 10	33	18	15	0	0,0	0	0,0	0	0,0	0	0,0	3	16,7	3	20,0	3	16,7	3	20,0
10 < 11	32	9	23	0	0,0	0	0,0	0	0,0	0	0,0	2	22,2	4	17,4	1	11,1	9	39,1
11 < 12	54	24	30	0	0,0	0	0,0	1	4,2	4	13,3	6	25,0	2	6,7	0	0,0	0	0,0
12 < 13	49	25	24	0	0,0	0	0,0	1	4,0	2	8,3	3	12,0	2	8,3	0	0,0	0	0,0
13 < 14	49	22	27	0	0,0	0	0,0	0	0,0	0	0,0	1	4,5	9	33,3	0	0,0	0	0,0
14 < 15	79	41	38	1	2,4	0	0,0	2	4,9	1	2,6	2	4,9	8	21,1	1	2,4	0	0,0
15 < 16	74	40	34	1	2,5	0	0,0	5	12,5	4	11,8	1	2,5	4	11,8	0	0,0	1	2,9
16 < 17	43	20	23	0	0,0	0	0,0	2	10,0	1	4,3	0	0,0	0	0,0	0	0,0	0	0,0
17 < 18	44	32	12	0	0,0	0	0,0	3	9,4	1	8,3	0	0,0	3	25,0	0	0,0	0	0,0
18 < 19	34	17	17	0	0,0	0	0,0	0	0,0	0	0,0	0	0,0	2	11,8	0	0,0	0	0,0

Total	595	306	289	20	6,5	16	5,5	15	4,9	14	4,8	19	6,2	38	13,1	17	5,6	18	6,2

					Total cases				Total cases				Total cases				Total cases		

					*n*. 36	6,1%			*n*. 29	4,9%			*n*. 57	9.6%			*n*. 35	5.9%	
